# ENHANZE^®^ drug delivery technology: a novel approach to subcutaneous administration using recombinant human hyaluronidase PH20

**DOI:** 10.1080/10717544.2018.1551442

**Published:** 2019-02-11

**Authors:** Kenneth W. Locke, Daniel C. Maneval, Michael J. LaBarre

**Affiliations:** Halozyme Therapeutics, Inc., San Diego, CA, USA

**Keywords:** Recombinant human hyaluronidase PH20, rHuPH20, hyaluronan, ENHANZE, subcutaneous

## Abstract

ENHANZE^®^ drug delivery technology is based on the proprietary recombinant human hyaluronidase PH20 enzyme (rHuPH20; Halozyme Therapeutics, Inc.) that facilitates the subcutaneous (SC) delivery of co‐administered therapeutics. rHuPH20 works by degrading the glycosaminoglycan hyaluronan (HA), which plays a role in resistance to bulk fluid flow in the SC space, limiting large volume SC drug delivery, dispersion, and absorption. Co-administration of rHuPH20 with partner therapies can overcome administration time and volume barriers associated with existing SC therapeutic formulations, and has been shown to reduce the burden on patients and healthcare providers compared with intravenous formulations. rHuPH20 (as HYLENEX^®^ recombinant) is currently FDA-approved for subcutaneous fluid administration for achieving hydration, to increase the dispersion and absorption of other injected drugs, and in subcutaneous urography for improving resorption of radiopaque agents. rHuPH20 is also co-formulated with two anticancer therapies, trastuzumab (i.e. Herceptin^®^ SC) and rituximab (i.e. RITUXAN HYCELA^®^/RITUXAN^®^ SC/MabThera^®^ SC) and dosed sequentially with human immunoglobin to treat primary immunodeficiency (i.e. HyQvia^®^/HYQVIA^®^). This article reviews pharmaceutical properties of rHuPH20, its current applications with approved therapeutics, and the potential for future developments.

## Introduction

**Figure 1.**
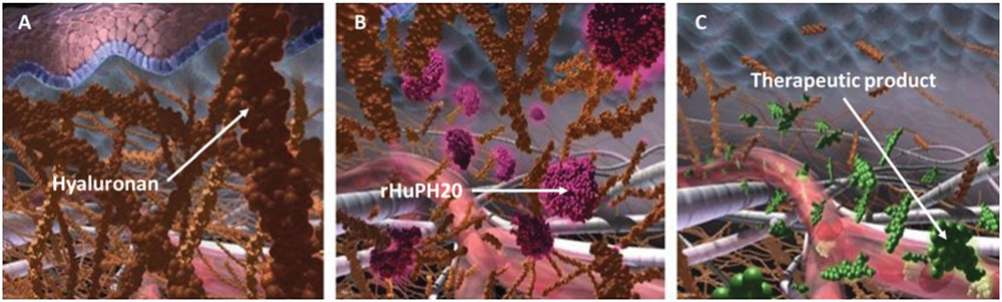
rHuPH20 mechanism of action. (A) Hyaluronan creates a resistance to bulk fluid flow and limits large volume SC drug delivery, dispersion, and absorption. (B) rHuPH20 depolymerizes hyaluronan, (C) facilitating SC bulk fluid flow and increasing the dispersion and absorption of co-administered therapeutics.

ENHANZE^^®^^ drug delivery technology utilizes a proprietary recombinant human hyaluronidase PH20 (rHuPH20; Halozyme Therapeutics, Inc.) to facilitate the subcutaneous (SC) delivery of co‐administered therapies. rHuPH20 works by locally degrading hyaluronan (HA), a large glycosaminoglycan and component of extracellular, pericellular, and intracellular matrices (Frost, 2007; Shepard, 2015). Hyaluronan is a key component of the skin that forms a gel-like substance with water, creating resistance to bulk fluid flow and limiting large volume SC drug delivery, dispersion, and absorption (Figure 1) (Frost, 2007).

Hyaluronidases as a class have been used for the last 70 years to modify tissue permeability through degradation of HA (Atkinson, 1949). rHuPH20 specifically has been shown to facilitate SC bulk fluid flow and to increase the dispersion and absorption of co‐administered therapeutics (Bookbinder et al., 2006; Thomas et al., 2009a; Vaughn & Muchmore, 2011; Wasserman, 2012; Dychter et al., 2014). rHuPH20 can enable intravenously administered therapies to be given subcutaneously and permits larger SC administration volumes that can reduce the number (and potential frequency) of individual injections required. Furthermore, clinical studies have shown that rHuPH20 has the potential to improve the pharmacokinetic (PK) profiles of co-administered SC agents compared with SC administration without rHuP20; it can potentially increase the absorption rate, increase bioavailability, increase maximum plasma concentrations (*C*_max_), accelerate time to reach maximum concentration (*T*_max_), and decrease intra-individual variability in PKs (Thomas et al., 2009b; Vaughn et al., 2009; Morrow et al., 2011; Wasserman et al., 2012).

The action of rHuPH20 in the SC space has been shown to be local and transient; within 24 hours of injection, normal interstitial viscoelasticity is restored without inflammatory changes or histologic alterations (Bookbinder et al., 2006). rHuPH20 is generally well tolerated with the most frequently reported adverse events (AEs) being mild injection-site reactions (U.S. Food and Drug Administration, 2005; Connolly et al., 2011). The AE profile of rHuPH20 and co-administered therapeutics generally reflects that of the individual agents (Wasserman, 2014a; Wasserman et al., 2016a) or relates to the rapid introduction of fluid into the SC space (e.g. mild-to-moderate infusion site swelling and edema) (Thomas et al., 2009a; Spandorfer et al., 2012; Dychter et al., 2014; Wasserman, 2014a). Anti-rHuPH20 antibodies have been detected, but typically occur infrequently and have not been associated with AEs; in addition, no neutralizing antibodies have been reported (Rosengren et al., 2015).

Application of rHuPH20 as part of the ENHANZE drug delivery technology may overcome administration time and volume barriers associated with existing SC therapeutic formulations, and has been shown to reduce the burden on patients and healthcare providers compared with intravenous formulations. These changes may increase health system efficiencies by reducing healthcare provider time and the use of facilities such as infusion chairs to administer IV infusions (Burcombe et al., 2013; Garrun, 2013; Pivot et al., 2014; De Cock et al., 2016), and may also facilitate improved patient experiences (Pivot et al., 2014; Rummel et al., 2017). Furthermore, application of rHuPH20 may enable reductions in the amount of drug product required by increasing the bioavailability of SC formulations (Wasserman et al., 2012). This article reviews the properties of rHuPH20, its current applications with approved therapeutics, and potential future developments.

## Development of rHuPH20

The activity of hyaluronidases as a "spreading factor" that increases the dispersion of injected substances in tissues was initially discovered from experiments using extracts of rabbit testes in the late 1920s (Duran-Reynals, 1928, 1933). However, it was not until the late 1930s that this factor was purified and further characterized as the enzyme hyaluronidase (Chaik & Duthie, 1939).

The hyaluronidases are a family of enzymes, including Hyal-1 and Hyal-2, which are responsible for much of the HA degradation in the body, and sperm adhesion molecule 1 (SPAM1, also referred to as PH20), which is located on the surface of sperm and degrades HA to facilitate sperm penetration of the oocyte during fertilization (Lin et al., 1994; Csoka et al., 2001; Chao et al., 2007). Of the hyaluronidases, PH20 is unique in that it shows enzymatic activity at both acidic and neutral pH (Cherr et al., 2001). Most mammalian hyaluronidases, including PH20, break down HA by hydrolysis of the β-1,4 glycosidic bond into various oligosaccharides, the shortest of which are tetrasaccharides (Stern & Jedrzejas, 2006).

Animal-derived PH20 hyaluronidases (bovine and ovine) have been used to increase the absorption and dispersion of other injected drugs or fluids since the 1940s (Atkinson, 1949). As a group, hyaluronidases have been the subject of multiple investigations and regulatory approvals globally. It is estimated that tens of millions of doses have been administered to humans (U.S. Food and Drug Administration, 2003). However, animal-derived hyaluronidase formulations have been associated with allergic reactions, including anaphylaxis (Baumgartner et al., 1998; Dunn et al., 2010). In contrast, rHuPH20 has not been associated with the allergic and immunogenic problems associated with the nonhuman, animal-derived preparations (Baumgartner et al., 1998; Yocum et al., 2007). The replacement of animal-derived products with recombinant human products is recommended by the World Health Organization wherever possible because of concern about the transmission of Creutzfeldt-Jakob disease (World Health Organization, 2003).

rHuPH20, the proprietary enzyme developed by Halozyme, is a purified recombinant human form of PH20. rHuPH20 has been shown to be pure and potent *in vitro* (approximately 140- to 200-fold increase compared with compounded animal-derived hyaluronidase; approximately 5.6-fold increase compared with manufactured animal-derived hyaluronidase) (Silverstein et al., 2012). rHuPH20’s mechanism of action has been demonstrated in a number of preclinical studies using immunoglobulin G (IgG) as a representative therapeutic protein (Kang et al., 2013). These studies, in which minipigs were used as a model for human skin, confirmed that SC delivery of rHuPH20 increased the dispersion and absorption of large volumes of co-administered therapeutic proteins (Kang et al., 2013). Compared with control infusions, rHuPH20 significantly reduced infusion pressure and induration and accelerated postinfusion IgG dispersion.

In addition to the extensive clinical experience with animal-derived hyaluronidases and their regulatory approvals confirming the utility of the approach, rHuPH20 has been studied in a comprehensive program of clinical trials undertaken by Halozyme, including 28 studies conducted under the HYLENEX^^®^^ investigational new drug application (IND) or as postmarketing, non-IND studies. In these studies, individual doses of rHuPH20 ranged from 15 to 96,000 U (data on file). The completed studies demonstrated the facilitation of SC fluid administration, as well as improved delivery of small molecules (e.g. ceftriaxone, morphine), insulin and insulin analogs, and proteins (e.g. IgG and/or adalimumab), in terms of larger injection volumes, increased bioavailability and *C*_max_, and faster *T*_max_ compared with SC delivery without rHuPH20 (Frost, 2007; Thomas et al., 2009b; Vaughn et al., 2009; Morrow et al., 2011; Wasserman et al., 2012). For insulin analogs, rHuPH20 co-injection reduced intra-individual pharmacokinetic variability (Morrow et al., 2011). In addition, the “faster in/faster out” profile has been shown to result in more rapid onset and offset of insulin action (Bookbinder et al., 2006; Frost 2007; Morrow et al., 2011, 2013).

Subcutaneous injections of rHuPH20 in combination with hydration fluids, co-injected drugs and biologic products were generally well tolerated in all clinical study populations, including healthy subjects, dehydrated pediatric subjects, hospice and palliative care subjects, subjects with type 1 and 2 diabetes mellitus, and subjects with rheumatoid arthritis. Most AEs were mild, transient injection-site reactions, including erythema, pain, bruising, pruritus, burning, tenderness, edema, induration, irritation, paresthesia, numbness, and rash. Moderate injection-site reactions, which occurred less frequently, include burning, erythema, pain, and numbness. Mild-to-moderate headache was also commonly reported. Adverse events have otherwise generally reflected the adverse reaction profiles of the co-administered drug or have been associated with the rapid introduction of a relatively large volume of fluid into the SC space (data on file). As the tissue changes induced by rHuPH20 are reversible within 24 h after each administration without any documented inflammatory or histological changes (Bookbinder et al., 2006), lasting changes of the SC space are not expected with long-term use of rHuPH20.

Hyaluronidase human injection (HYLENEX^®^ recombinant; rHuPH20) has been available since 2005 in the US and is indicated as an adjuvant: in SC fluid administration for achieving hydration; to increase the dispersion and absorption of other injected drugs; and in SC urography for improving resorption of radiopaque agents (U.S. Food and Drug Administration, 2005). Based on the number of vials sold to date and assuming one vial per patient, rHuPH20 has been administered to nearly 2 million patients as HYLENEX recombinant (data on file).

According to the US prescribing information, HYLENEX recombinant (150 U) can be injected prior to the start of subcutaneous fluid administration to facilitate absorption of 1000 mL or more of solution (U.S. Food and Drug Administration, 2005). The dose, rate of injection, and type of solution need to be adjusted on an individual basis. Hypovolemia can be avoided by using solutions containing adequate amounts of inorganic electrolytes and/or controlling the volume and speed of administration. HYLENEX recombinant may also be added to small volumes of fluid replacement solutions or solutions of drugs for SC injection, with specific fluid dosage dependent upon age, weight, clinical condition and laboratory parameters. The dispersion and absorption of other injected or SC infused drugs can also be enhanced by pre-administration of HYLENEX recombinant or by adding 50–300 U (typically 150 U) hyaluronidase to the injection solution prior to infiltration, interstitial, intramuscular, intraocular, retrobulbar, soft tissue or SC use. Finally, HYLENEX recombinant may also be used to facilitate SC administration of urographic contrast media when IV administration is difficult to achieve, particularly in infants and small children. (U.S. Food and Drug Administration, 2005).

## Application of rHuPH20 in marketed products

**Figure 2.**
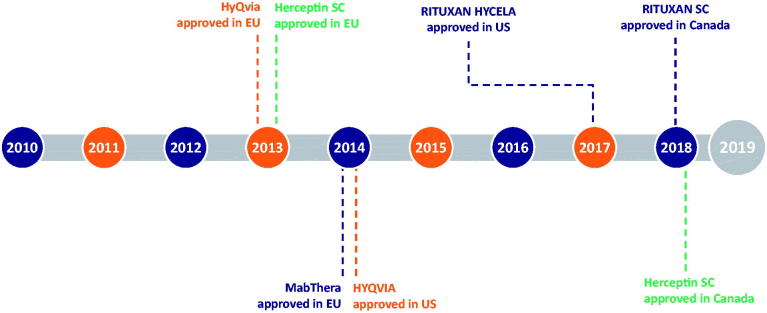
Timeline of rHuPH20 partner product approvals. HyQVIA/HYQVIA, human immunoglobulin infusion (Immune Globulin 10%) and rHuPH20; Herceptin SC, trastuzumab and rHuPH20; MabThera SC/RITUXAN HYCELA/RITUXAN SC, rituximab and rHuPH20. Rituximab and trastuzumab were the first and second monoclonal antibodies approved for the treatment of cancer, respectively (Dillman,^1999^; Pierpont et al.,^2018^), and the first to undergo IV to SC conversion.

In addition to HYLENEX^®^ recombinant, rHuPH20 is approved in more than 50 countries for use in co-formulations with two different anticancer therapies, trastuzumab and rituximab (marketed as Herceptin^®^ SC and RITUXAN HYCELA^®^/RITUXAN^®^ SC/MabThera^®^ SC, respectively), and is administered sequentially with human immunoglobin for primary immunodeficiency (marketed as HyQvia^®^/HYQVIA^®^). Figure 2 shows the timeline of rHuPH20 partner product approvals.

### HYQVIA

As part of a combination therapy, rHuPH20 was first approved in 2013 in the EU for use with human immunoglobulin infusion (IgG [immune globulin 10%]) for the treatment of primary immunodeficiency (PI) as HyQvia (Shire Pharmaceuticals) (European Medicines Agency, 2013a). Approval followed in the US in 2014 under the name HYQVIA (U.S. Food and Drug Administration, 2012).

Primary immunodeficiency (PI) is characterized by absent or deficient antibody production and typically results in recurrent and/or severe infections (Perez et al., 2017). The condition carries a high treatment burden for patients as immunoglobulin replacement is required for life and usually requires monthly or weekly replacement treatments, depending on the mode of administration. Typical IgG doses for PI range from 300–600 mg/kg (150–600 mL/month), and traditional delivery options have been either monthly intravenous IgG infusions (IGIV) or weekly SC administration at multiple injection sites (2–20 sites/month) to accommodate the large injection volume (Wasserman et al., 2016a). Individualized dosing is recommended for both standard IV and SC forms of IgG, and a wide range of dosing schedules are employed by immunologists in the United States and Europe (Perez et al., 2017).

With HYQVIA, the full monthly dose of IgG (up to 600 mL) may be delivered using a single SC injection site (rHuPH20 followed sequentially by IgG), which allows most patients to be dosed every 3–4 weeks rather than requiring multiple weekly injections to deliver the monthly IgG SC dose without rHuPH20 (European Medicines Agency, 2013b; U.S. Food and Drug Administration, 2014). HYQVIA may also be self-administered in the home setting (Wasserman, 2014b). rHuPH20 has also been shown to increase the bioavailability of IgG by 20% compared with other SC IgG products (Wasserman, 2012). The bioavailability of IgG when given sequentially after rHuPH20 has been shown to be pharmacokinetically equivalent to that of IGIV (93.3% of IGIV), when comparing area under the plasma concentration-time curve (AUC); no dose adjustment from IV is necessary (U.S. Food and Drug Administration, 2014).

HYQVIA demonstrates similar efficacy and tolerability to IGIV, but with fewer systemic reactions than IGIV (8.3% vs. 25.0%) (Wasserman et al., 2012). The most common AEs associated with HYQVIA were local reactions at the infusion site similar to those seen with other SC IgGs (Wasserman et al., 2012). Because the HYQVIA product requires chronic dosing, an understanding of potential adverse effects associated with repeat-dosing of rHuPH20 was warranted. In Good Laboratory Practice (GLP)-compliant repeat-dose toxicity studies conducted in cynomolgus monkeys, rHuPH20 was well tolerated following once weekly SC administration (up to 39 weeks), and there were no adverse effects at the highest doses tested (Baxter BioScience, 2014) (data on file). In addition, GLP-compliant embryo-fetal development and pre-/post-natal development studies were conducted in CD-1 mice (Baxter BioScience, 2014) (data on file). While rHuPH20 was embryo-fetotoxic at ≥9 mg/kg/dose (>10,000 times higher than the typical monthly human dose), it did not demonstrate teratogenic potential. As anti-rHuPH20 antibodies have the potential to cross-react with endogenous PH20, a series of developmental and reproductive toxicity (DART) studies were conducted in New Zealand White rabbits to comprehensively characterize the potential effect of anti-rHuPH20 antibodies on endogenous PH20 activity. The presence of anti-rHuPH20 antibodies did not demonstrate any adverse effects on male and female fertility, embryo-fetal development, or offspring development in the DART studies (Baxter BioScience, 2014; Veneziale et al., 2014) (data on file). Furthermore, a series of non-GLP investigational studies of gene expression of SPAM1/PH20 in mouse, rabbit, and human tissues confirmed that PH20 is primarily expressed in the male reproductive tract (Baxter BioScience, 2014; Veneziale et al., 2014) (data on file). The comprehensive nonclinical data package supporting the safe use of rHuPH20 by the SC route of administration in humans was included in the HYQVIA EMA and US FDA submissions.

The long-term safety of HYQVIA has also been studied in humans. An analysis of two Phase 3 studies found long-term treatment with HYQVIA to be well tolerated in both adults and children (Wasserman et al., 2016b). That analysis looked at data from 188 patient-years of HYQVIA exposure, with exposures of longer than 30 months in 48 patients. The incidence of both systemic and local AEs was low and the rate of local AEs declined during the treatment course. These findings provide reassurance that long-term HYQVIA use is well tolerated and does not appear to be associated with AEs related to length of treatment.

HYQVIA may be a more cost-efficient option compared with some other SC IgG products due to its ∼20% increase in bioavailability, and by removing the need for multiple weekly injections. Reducing the frequency of treatment can alleviate the burden associated with each SC administration, such as the scheduling of appointments, preparation of the materials for SC infusion and preparation of the infusion site, safe disposal of used materials, and documentation of administration, whether carried out by the patient themselves or a healthcare provider (Great Ormond Street Hospital, 2017). Fewer SC administrations also reduce the costs of the disposable materials required for administration (e.g. syringes, needles), which may not be insignificant when considered over a lifetime of treatment for PI. In addition, HYQVIA may be infused over a shorter period of time than conventional SC IgGs, which may further reduce the burden on patients and healthcare providers. Based on the combined effects of fewer administrations, shorter administration periods, and increased bioavailability, it may be reasonable to postulate that HYQVIA may be more cost-effective than other SC IgGs.

### Herceptin SC

Herceptin SC (Roche Products Ltd) combines rHuPH20 and trastuzumab, a monoclonal antibody that targets human epidermal growth factor receptor 2 protein (HER2). The combination of rHuPH20 (2000 U/mL) and trastuzumab (600 mg/5 mL) is approved for SC administration for the treatment of HER2-positive early breast cancer, metastatic breast cancer, and metastatic gastric cancer in the EU (European Medicines Agency, 2018b), for the treatment of HER2-positive early and metastatic breast cancer in Canada (Roche Canada, 2018a), and is under review by the FDA for use in the US (Biologics License Application accepted in July 2018 for Herceptin SC; (Halozyme Therapeutics Inc., 2018).

With the SC formulation, the trastuzumab dose can be administered in ≤5 min rather than over 30–90 min as an IV infusion (European Medicines Agency, 2018b). Moreover, the SC formulation is provided at a fixed dose of 5 mL containing 600 mg of trastuzumab for all patients and removes the need for a loading dose and the weight-based dosing required for the IV formulation (European Medicines Agency, 2018b).

In clinical studies, SC trastuzumab demonstrated similar systemic exposure and safety to the IV formulation (Wynne et al., 2013). In healthy volunteers, fewer AEs have been observed with the SC formulation compared with the IV formulation at all dose levels investigated, including fewer infusion-related reactions. However, local administration site AEs (injection-site discoloration, discomfort, erythema, pain, reaction, and swelling) were more common with SC delivery (Wynne et al., 2013). In a Phase 3 study, the overall incidence of AEs, including grade 3 or 4 AEs, was similar between SC and IV treatment groups (Jackisch et al., 2015). There was a slightly higher incidence of serious AEs in the SC group consisting mainly of infections, but these events were rare and the differences were small (Jackisch et al., 2015). This study also showed that the pathologic complete response rate was comparable between the SC and IV formulations of trastuzumab (Jackisch et al., 2015). The SC formulation of trastuzumab may also enable time and healthcare cost savings, as the change from IV to SC formulation has been shown to reduce both patient chair and active healthcare provider times versus IV infusion (De Cock et al., 2016). Mean time savings per session were 55–57 min of patient chair time and 13–17 min of active healthcare provider time with SC trastuzumab (delivered via a single-use injection device or a hand-held syringe) versus IV infusion (De Cock et al., 2016). In a UK study, cost savings associated with SC versus IV trastuzumab have been estimated as £111.81 per patient episode, equating to a potential saving of £2012.58 per patient over a full course of adjuvant trastuzumab treatment for HER2-positive early breast cancer (Burcombe et al., 2013). In addition, patients have indicated a significant preference for SC trastuzumab over IV infusion, whether delivered via a single-use injection device or a hand-held syringe (Pivot et al., 2014).

### RITUXAN HYCELA^^®^^/RITUXAN^^®^^ SC/MabThera^^®^^ SC

Rituximab (Roche) is a chimeric murine/human monoclonal antibody that binds to cluster of differentiation 20 (CD20) protein present on the surface of pre-B- and mature B-lymphocytes (European Medicines Agency, 2018b). Rituximab, in combination with rHuPH20, has the following approvals: in the EU as a solution for SC administration in the treatment of certain types of non-Hodgkin’s lymphoma (follicular lymphoma and CD20-positive diffuse large B-cell lymphoma) as MabThera SC (European Medicines Agency, 2018c); in the US for chronic lymphocytic leukemia (CLL), follicular lymphoma, and diffuse large B-cell lymphoma as RITUXAN HYCELA (U.S. Food and Drug Administration, 2017c); and, in Canada for non-Hodgkin’s lymphoma and CLL as RITUXAN SC (Roche Canada, 2018b).

Rituximab SC has been characterized in a series of clinical studies that demonstrated non-inferiority in systemic exposure with similar efficacy and safety to that of the IV formulation (Davies et al., 2014; Salar et al., 2014; Assouline et al., 2016), as well as compelling patient preference for the SC formulation over rituximab IV (Rummel et al., 2017). Indeed, both serum trough concentrations (*C*_trough_) and the AUC for rituximab SC were found to be non-inferior to those of rituximab IV (Davies et al., 2014; Salar et al., 2014; Assouline et al., 2016). In studies in patients with follicular lymphoma or CLL, comparable B-cell depletion was also observed throughout treatment with rituximab IV and SC (Davies et al., 2014; Salar et al., 2014; Assouline et al., 2016) and there was evidence of B-cell repletion 9 months after the last rituximab (IV or SC) administration (Salar et al., 2014; Assouline et al., 2016). Rituximab SC also demonstrated comparable efficacy and safety with the IV formulation in three randomized controlled studies of patients with B-cell malignancies (Assouline et al., 2016; Davies et al., 2017; Lugtenburg et al., 2017).

Rituximab SC may have advantages for both patients and clinicians compared with IV delivery, including fixed versus patient-specific (body surface area-based) dosing, a ready-to-use vial, and substantially reduced administration times (5–7 min vs. 1.5–2.5 h) (U.S. Food and Drug Administration, 2017a, 2017d; European Medicines Agency, 2018b). In a UK study, rituximab SC saved an average of 174.8 min of total active healthcare provider time per session compared with the IV formulation (Rule et al., 2014). Patient time in the treatment room was also reduced from 263.8 min for IV rituximab to 70.0 min for SC rituximab, per session. In addition, SC rituximab reduced total mean staff costs by £115.17 per session compared with rituximab IV (Rule et al., 2014). In a study of patients with untreated CD20+ diffuse large B-cell lymphoma or follicular lymphoma, 81% preferred rituximab SC over rituximab IV and patient satisfaction was higher with the SC formulation over the IV formulation (Rummel et al., 2017).

Rituximab SC is approved for use at two doses: 1400 mg per 11.7 mL (Europe, US and Canada) and 1600 mg per 13.4 mL (US and Canada) (U.S. Food and Drug Administration, 2017c; Roche Canada, 2018b; European Medicines Agency, 2018b); the SC formulations of rituximab contain 2000 U/mL rHuPH20. The approval of rituximab SC in the US (three indications and two dose levels, as described previously) was based primarily on the basis of a PK-bridging approach to establish the safety and effectiveness of RITUXAN HYCELA (rituximab and rHuPH20) for SC administration (U.S. Food and Drug Administration 2017a, 2017b). Although the PK-bridging clinical trials were not designed for efficacy hypothesis testing, the numerical efficacy results were comparable between rituximab SC and IV. The US FDA requested discussion at an Oncologic Drugs Advisory Committee (ODAC) meeting to obtain feedback and insights on the acceptability of the above development approach to support the approval of rituximab SC for the same oncologic indications as rituximab IV (RITUXAN); the approval, as recommended by the ODAC, was subsequently based on the comparative PK assessments provided (U.S. Food and Drug Administration, 2017b).

## ENHANZE^^®^^ drug delivery technology

ENHANZE drug delivery technology combines rHuPH20 and Halozyme’s expertise in SC delivery. At the core of the ENHANZE drug delivery technology is rHuPH20, which facilitates the route of administration changes, enables IV therapies to be administered SC, and can optimize SC dosing. The other components of ENHANZE drug delivery technology offer support with broader aspects of partner product development, as described in this section.

### ENHANZE™ drug product (EDP)

EDP is an investigational product that allows co-mixing of the partner therapeutic with rHuPH20 at the clinical site (simulating a co-formulated product) to allow quick initiation of early stage trials. This can shorten the time to entry into dose-finding Phase 1 trials compared with developing a co-formulation (single vial) prior to the initiation of clinical trials. Co-formulation efforts generally occur in parallel with the early trials to support pivotal Phase 3 studies.

### Expertise in SC delivery, clinical development, and regulatory strategy

Traditional medical thinking suggests that SC injections are typically limited to small volumes (e.g. <2 mL) to avoid pain and induration at the injection site. As shown in preclinical and clinical studies, rHuPH20 removes this barrier to SC drug administration. An in-depth understanding of SC delivery (in terms of dose administration, flow rates, needle, and tubing sizes), nonclinical porcine models and PK modeling at Halozyme has helped to facilitate the development of partner SC products. For example, understanding the critical relationships of viscosity and system hardware (e.g. Hagen-Poiseuille Law (Sutera & Skalak, 1993)) to fluid flow rate has enabled Halozyme partners to optimize the SC delivery of their products. Moreover, during the development of the approved partner products, it has been possible to gain a greater understanding of the necessary clinical and regulatory requirements to streamline development strategy, as exemplified by the comparative PK noninferiority endpoints with RITUXAN HYCELA (U.S. Food and Drug Administration, 2017b).

## Products under development

In addition to the above approved products, rHuPH20 is in clinical development with several approved IV therapeutics and investigational products. The most advanced of these developmental partnerships is with daratumumab (DARZALEX^®^; Janssen), the first approved monoclonal antibody for the treatment of multiple myeloma targeting CD38, an antigen highly expressed on multiple myeloma cells. The daratumumab and rHuPH20 co-formulated product has entered Phase 3 clinical trials. The development timeline for the daratumumab and rHuPH20 co-formulation has been accelerated through the use of EDP. That is, the use of EDP with daratumumab for multiple myeloma enabled Phase 1 trials of the SC formulation to start within 8.5 months of partnership kickoff, and initiation of Phase 3 trials (using a co-formulated drug product) less than 24 months from filing of the IND application. Development of the co-formulated product (single vial) for use in Phase 3 trials, and ultimately for the market, occurred in parallel with the Phase 1 studies. Results from a Phase 1 study (NCT02519452) demonstrated that EDP-enabled daratumumab significantly reduced infusion time (from 3.4 to 7 h IV to <5 min SC) and reduced the rate of infusion-site reactions versus the IV formulation by ∼75% (Chari et al., 2017; European Medicines Agency, 2018a). The ongoing open-label Phase 3 trial (NCT03277105) aims to demonstrate non-inferiority of daratumumab SC to daratumumab IV in terms of overall response rate and plasma *C*_trough_ daratumumab levels in patients with multiple myeloma. Final results from this trial are expected in 2020.

In addition, Alexion Pharmaceuticals is using the ENHANZE drug delivery technology to develop a next-generation SC formulation of ALXN1210 (ALXN1210 + rHuPH20, designated as ALXN1810), an investigational long-acting C5 complement inhibitor monoclonal antibody in development for the treatment of paroxysmal nocturnal hemoglobinuria (Alexion Pharmaceuticals I, 2017, 2018). Bristol-Myers Squibb is also undertaking a global collaboration with Halozyme to develop subcutaneously administered immuno-oncology medicines using the ENHANZE drug delivery technology. Included among those medicines is nivolumab (OPDIVO^®^), which is currently approved in the US to treat 10 types of cancer via a 30-min IV infusion.

## Summary

ENHANZE drug delivery technology is based on the action of rHuPH20, a proprietary purified recombinant human hyaluronidase PH20. Hyaluronidases have been used to increase the absorption and dispersion of injected drugs or fluids since the 1940s. rHuPH20 facilitates route of administration changes (enabling IV therapies to be administered subcutaneously) and can optimize the dosing of SC therapies. Co-administration of rHuPH20 with partner products can overcome administration time and volume barriers associated with existing therapeutic formulations and may reduce the burden on patients and healthcare providers compared with IV formulations. Moreover, rHuPH20 minimizes the allergenicity and immunogenicity associated with the animal-derived hyaluronidases. rHuPH20 is currently FDA-approved to increase the absorption and dispersion of SC drugs (HYLENEX recombinant) and is available in various territories in combination with three approved partner products (HyQvia/HYQVIA, Herceptin SC, and RITUXAN HYCELA/RITUXAN SC/MabThera SC).
